# Chylous ascites associated with chylothorax; a rare sequela of penetrating abdominal trauma: a case report

**DOI:** 10.1186/1752-1947-1-149

**Published:** 2007-11-25

**Authors:** Joseph M Plummer, Michael E McFarlane, Arhcibald H McDonald

**Affiliations:** 1 Department of Surgery, Radiology, Anaesthesia and Intensive Care, University of the West Indies, Kingston 7, Jamaica

## Abstract

We present the case of a patient with the rare combination of chylous ascites and chylothorax resulting from penetrating abdominal injury. This patient was successfully managed with total parenteral nutrition. This case report is used to highlight the clinical features and management options of this uncommon but challenging clinical problem.

## Introduction

Although traumatic chylous ascites was first described in the 17^th ^century by Morton [1] fewer than 100 cases have been reported in the world literature [2]. We recently managed a patient with chylous ascites resulting from penetrating trauma and who developed a right-sided chylous pleural effusion during the course of his treatment. This is the only case of combined chylous ascites and chylous pleural effusion resulting from penetrating trauma that we are aware of in the English medical literature. The management of this rare but potentially debilitating condition is discussed.

## Case presentation

A 19-year-old male was seen by the surgical team 14 hours after suffering a gunshot wound to the upper abdomen. On examination he was haemodynamically normal but he had a right pneumothorax for which a thoracostomy tube was inserted. His abdomen was distended with an entry gun-shot wound in the epigastrium four centimeters to the left of the midline and exit gun-shot wound posteriorly on the right at the level of the twelfth thoracic vertebra, eight centimeters from the midline. Neurological examination revealed lower limb paresis but there was no sensory deficit. Plain x-rays revealed full expansion of the lungs and a comminuted fracture to the lateral body of the T_12 _vertebra and the associated twelfth rib.

He underwent mandatory exploratory laparotomy, which revealed 3.0 litres of blood, haemoperitoneum and a liver injury to segment four which was not actively bleeding. A small amount of clear fluid was noted to be accumulating in the retroperitoneum of the upper abdomen but its origin was unclear.

His thoracostomy tube was removed and he was discharged five days after the laparotomy. The management plan for his vertebral fracture was non-operative with a brace and bed rest.

The patient re-presented three weeks later with painless abdominal distension and shortness of breath. There was no history of vomiting or constipation. Examination of the abdomen revealed a non-tender distended abdomen with ascites which was confirmed on ultrasound. Erect chest radiograph was normal. A diagnostic and therapeutic abdominal paracentesis was performed. Five liters of milky white fluid was obtained. Chemical analysis was as follows – triglycerides 13.5 mmol/L, cholesterol 1.3 mmol/L, amylase 28 IU/L, and total protein 56 g/L with albumin of 37 g/L. Culture of the aspirate revealed no growth. A diagnosis of traumatic chylous ascites was made based on the physical appearance of the fluid and the cholesterol: triglyceride ratio of less than one. His management consisted of nil by mouth, total parenteral nutrition (TPN) and frequent abdominal paracentesis, which was performed on five occasions removing a total of 20.0 liters. Ten days after re-admission he was diagnosed with a right pleural effusion after developing dyspnea. Aspiration of the pleural fluid also revealed chyle which was confirmed by its chemical analysis which was identical to the peritoneal aspirate. This required thoracocentesis to control his shortness of breath and a total of four liters was aspirated.

Total parenteral nutrition was administered for a total of five weeks. He was gradually established on a normal diet. Both ultrasound and chest x-ray were normal eight weeks after commencing treatment. He also experienced good improvement in his neurological function and was discharged for outpatient follow-up.

## Discussion

Chylous ascites is the accumulation of extravasated chyle in the peritoneal cavity. Chylous ascites is milky in appearance and separates into layers upon standing. The concentration of triglycerides in chyle is higher than that of plasma while its cholesterol concentration is less than in plasma. This cholesterol: triglyceride ration of less than 1 is diagnostic of chyle [3].

The commonest cause of chylous ascites in adults is obstruction due to lymphomas and other malignancies, while in children congenital lesions of the visceral lymphatics predominate [4]. Trauma now accounts for approximately 20% of paediatric chylous ascites, with child abuse probably account for 10% of cases [5].

Traumatic chylous ascites most frequently develops from blunt trauma resulting in tears at the root of the small bowel mesentery [2]. Such a force is usually associated with multiorgan injury and isolated cases of injury to the cisterna chyli caused by penetrating injuries are rare [2]. In our patient the rupture of the cisterna chyli may have been due to a direct penetrating injury caused by the gunshot. This would account for the clear fluid accumulating in the lesser sac at laparotomy. We theorise that the development of the effusion resulted from passage of chyle through transdiaphragmatic lymphatic channels in a manner similar to Meigs syndrome even though a direct extension cannot be ruled out.

The clinical picture of a patient with chylous ascites is similar to that seen in this case. The presentation is insidious with gradual accumulation of fluid and increase in abdominal girth. As the abdominal distension progresses dyspnea, nausea and vague abdominal pain associated with paralytic ileus may occur. Hypovolumia from continued fluid loss may be compounded by hypoproteinemia which results in transcapillary fluid shifts. During prolonged chyle loss the body's reserves of protein, fats, vitamins and electrolytes are depleted [6].

Currently, four therapeutic options are recognized: an oral diet with medium chain triglycerides, TPN, venoperitoneal shunting, and exploratory laparotomy with direct ligation [2]. Limiting dietary intake of long-chain triglycerides, and supplementing the diet with medium-chain triglycerides, should theoretically decrease the lymphatic flow. In practice dietary manipulation is not effective on its own [2]. Total parenteral nutrition is effective in providing nutrition in patients with traumatic chylous ascites and with time the chylous peritoneal fistula usually heals [4]. It is associated with prolonged hospitalization as was evident in our reported case. It is also expensive and carries a risk of infection.

Case reports of successful management of chylous ascites with the use of LeVeen or Denver peritoneovenous shunts have been published [7,8]. They are not used for long term management as occlusion, infection and mild disseminated intravascular coagulation are all possible serious complications.

Surgical ligation is the most direct solution to the problem and any recognized lymphatic extravasation should be handled by suturing of the offending site and this gives good success [4]. Difficulty in identifying the source of the chylous leak at laparotomy is encountered in up to 50% of cases [9] but can be increased by ingestion of lipophilic dyes just before surgery or via a nasogastric tube during laparotomy [10]. In the elective setting lymphoscintigraphy is the preferred initial test to localize the damaged lymphatics and this also facilitates ligation [2].

## Conclusion

Patients with traumatic chylous ascites can have effective treatment at initial laparotomy. More commonly the patient's diagnosis is delayed. The majority of these patients can be safely managed by TPN over a variable period. Failure of medical management warrants progression to surgery after pre-operative localization tests.

## Competing interests

The author(s) declare that they have no competing interests.

## Authors' contributions

All three authors (JP, MM and AM) were integral in the management of the patient and each author actively participated in preparing and approved the final version of this manuscript.

## Consent

Written informed consent was obtained from the patient for publication of this manuscript.
